# Rejection-Focused Precision Medicine in Kidney Transplantation: Biology, Biomarkers, and Artificial Intelligence

**DOI:** 10.3390/life16040674

**Published:** 2026-04-15

**Authors:** Luis Ramalhete, Rúben Araújo, Miguel Bigotte Vieira, Emanuel Vigia, Cecília R. C. Calado, Anibal Ferreira

**Affiliations:** 1 Blood and Transplantation Center of Lisbon, Instituto Português do Sangue e da Transplantação, Alameda das Linhas de Torres, n° 117, 1769-001 Lisbon, Portugal; 2 NOVA Medical School, Faculdade de Ciências Médicas, Universidade NOVA de Lisboa, 1169-056 Lisbon, Portugal; 3 iNOVA4Health—Advancing Precision Medicine, Núcleo de Investigação em Doenças Renais, NOVA Medical School, Faculdade de Ciências Médicas, Universidade NOVA de Lisboa, 1169-056 Lisbon, Portugal; 4 LBS—Lisbon Business & Government School, Rua de São Bernardo, 34-A, 1200-427 Lisbon, Portugal; 5 Serviço de Nefrologia, Nova Medical School, Hospital Curry Cabral, Unidade Local de Saúde de São José, 1050-099 Lisbon, Portugal; 6 Centro Clínico Académico de Lisboa, 1169-024 Lisbon, Portugal; 7 Hepatobiliopancreatic and Transplantation Center, Curry Cabral Hospital, Unidade Local de Saúde de São José, R. da Beneficência 8, 1050-099 Lisbon, Portugal; 8 ISEL—Instituto Superior de Engenharia de Lisboa, Rua Conselheiro Emídio Navarro 1, 1959-007 Lisbon, Portugal; 9 Institute for Bioengineering and Biosciences (iBB), The Associate Laboratory Institute for Health and Bioeconomy-i4HB, Av. Rovisco Pais, 1049-001 Lisbon, Portugal

**Keywords:** kidney transplantation, kidney allograft rejection, antibody-mediated rejection, T cell–mediated rejection, biomarkers, artificial intelligence, machine learning, precision medicine

## Abstract

Chronic kidney disease is rising worldwide, and kidney transplantation remains the preferred modality of kidney replacement therapy. However, long-term graft survival continues to be limited by chronic alloimmune injury, particularly antibody-mediated rejection (ABMR) and its chronic active form. This narrative review synthesizes contemporary evidence on the immunopathogenesis, epidemiology, diagnosis, and management of kidney allograft rejection, with a deliberate focus on studies from the last five years and on United States and European cohorts. We summarize current concepts of T cell–mediated rejection (TCMR), ABMR, mixed and donor-specific antibody (DSA)–negative phenotypes, and the evolution of the Banff classification, highlighting how chronic active ABMR has emerged as a leading cause of death-censored graft loss. We then critically appraise the conventional diagnostic triad of creatinine/eGFR, DSA, and biopsy and review emerging tools, including donor-derived cell-free DNA, urinary chemokines such as CXCL9 and CXCL10, additional blood- and urine-based biomarkers, and biopsy transcriptomics. We also examine how artificial intelligence and machine learning may support digital pathology, multimodal risk prediction, and data integration, while recognizing the current challenges of biological interpretability, external validation, and clinical implementation. Finally, we propose a rejection-focused precision-medicine framework and outline key research gaps, including multicenter validation, trial-ready endpoints, and governance for AI-enabled pathways. Overall, the field is moving from isolated diagnostic signals toward integrated, biologically informed, and clinically actionable approaches to rejection detection and risk stratification.

## 1. Introduction

Kidney transplantation (KT) offers the best available treatment for most patients with end-stage kidney disease, yet it still faces a central clinical paradox: short-term outcomes have improved substantially, whereas long-term graft survival remains limited by late alloimmune injury. Chronic kidney disease remains one of the most prevalent non-communicable diseases worldwide, affecting nearly 800 million adults, and the demand for effective kidney replacement therapies continues to rise across both the United States and Europe [1,2,3,4,5,6,7].

Over the last three decades, advances in surgical techniques, donor management, and immunosuppressive therapy have markedly improved early post-transplant outcomes. Yet these gains have not solved the problem that now matters most: a substantial proportion of grafts are still lost late, largely because of chronic alloimmune injury rather than surgical or purely non-immunologic complications [3,5,6,7,8,9].

Within this spectrum of late injury, chronic active antibody-mediated rejection (caABMR) has emerged as one of the leading causes of death-censored graft loss in contemporary series. caABMR is now understood as a clinicopathologic consequence of persistent humoral alloimmunity, usually driven by donor-specific antibodies (DSAs), most prominently directed against donor human leukocyte antigen (HLA) molecules, and characterized by microvascular inflammation, transplant glomerulopathy, and progressive interstitial fibrosis and tubular atrophy. At the same time, T cell–mediated rejection (TCMR), mixed rejection, and DSA-negative phenotypes remain clinically important, particularly because they may overlap, evolve over time, and contribute to chronic graft injury in ways that are not fully captured by conventional diagnostic frameworks [8,10,11,12,13,14,15,16,17,18].

Despite intensive investigation and multiple off-label therapeutic approaches, there are still no regulatory-approved therapies for chronic or acute antibody-mediated rejection, and randomized trials have often been underpowered or inconclusive. This gap between better early control and limited long-term precision is one of the central unresolved problems in KT. Our understanding of rejection has evolved in parallel with the Banff classification, which remains the international standard for kidney allograft pathology. The 2019 Banff update refined the diagnostic thresholds for TCMR and antibody-mediated rejection (ABMR), clarified the role of microvascular inflammation, and underscored the prognostic weight of chronic lesions. More recently, the Banff 2022 Kidney Meeting Report reappraised microvascular inflammation and formally integrated biopsy-based transcriptomic signatures, while acknowledging that a significant proportion of biopsies with clear ABMR-like injury lack detectable circulating DSAs. These advances have improved diagnostic precision, but they have also exposed the limits of relying on conventional histology and static serologic measurements to describe a dynamic and mechanistically heterogeneous process [3,8,9,14,16,17,18,19,20,21].

In routine clinical care, serum creatinine, estimated glomerular filtration rate (eGFR), proteinuria, and DSA testing remain the backbone of graft monitoring in both European and US transplant programs. However, these markers are late or imperfect surrogates of injury. Serum creatinine is insensitive to early damage, proteinuria is nonspecific, and DSAs may be absent in a substantial minority of biopsies with histologic features of ABMR. As a result, clinicians are often trying to infer active rejection from signals that are delayed, incomplete, or biologically ambiguous [8,18,22,23].

This diagnostic gap has driven increasing interest in non-invasive and minimally invasive biomarkers that may detect alloimmune injury earlier and improve longitudinal risk stratification. Among these, donor-derived cell-free DNA (dd-cfDNA) has gained particular prominence, with recent meta-analyses showing associations with both TCMR and ABMR and multicenter data demonstrating incremental diagnostic value beyond standard monitoring. Urinary chemokines such as CXCL9 and CXCL10 have also emerged as promising markers of intragraft inflammation and may detect subclinical rejection before overt functional decline becomes apparent. In parallel, high-throughput transcriptomics and targeted molecular diagnostics are increasingly being incorporated into Banff deliberations and into the interpretation of diagnostically ambiguous biopsies [17,24,25,26,27,28,29,30,31,32,33].

Artificial intelligence (AI) and machine learning (ML) are also entering the field. Deep-learning approaches have shown promise in digital pathology, lesion quantification, and rejection phenotyping, while ML-based prognostic models may improve prediction of rejection risk and graft survival beyond traditional statistical approaches. More broadly, AI has been proposed to integrate histology, serology, biomarkers, and clinical data into more individualized transplant pathways. Yet the growing number of tests and models does not automatically translate into precision medicine. Many emerging biomarkers capture tissue injury or immune activation without fully resolving the underlying mechanism, and many AI tools remain insufficiently validated or difficult to integrate into routine clinical workflows [34,35,36,37,38,39,40].

In this narrative review, we examine kidney allograft rejection through three connected lenses: biology, biomarkers, and artificial intelligence. We revisit the immunologic and pathologic foundations of TCMR, ABMR, mixed rejection, and DSA-negative phenotypes; critically assess the strengths and limitations of current serologic and histologic frameworks; and review the emerging landscape of molecular diagnostics, non-invasive biomarkers, and AI-enabled tools that may support a more precise and clinically actionable approach to rejection detection, classification, monitoring, and, ultimately, prevention.

## 2. Methods of Literature Review

This article was designed as a narrative review focused on kidney allograft rejection as a major barrier to long-term graft survival. The review was structured around three interconnected domains: (i) rejection biology and immunopathogenesis, (ii) conventional and emerging biomarkers, and (iii) AI and ML approaches relevant to rejection detection, classification, monitoring, and risk stratification.

Targeted literature searches were performed in PubMed/MEDLINE and Scopus using combinations of terms related to KT and rejection, including “kidney transplantation”, “kidney allograft rejection”, “antibody-mediated rejection”, “T cell-mediated rejection”, “chronic active antibody-mediated rejection”, “donor-specific antibodies”, “Banff classification”, “donor-derived cell-free DNA”, “CXCL9”, “CXCL10”, “molecular diagnostics”, “transcriptomics”, “artificial intelligence”, “machine learning”, and “precision medicine”. Literature selection prioritized peer-reviewed studies published within the last five years, in keeping with the contemporary focus of this review, while earlier landmark articles were included when necessary to contextualize foundational concepts in transplant immunology, rejection classification, donor-specific antibodies, and biopsy- and molecular-based diagnostics.

Priority was given to original studies, multicenter cohorts, meta-analyses, consensus reports, and clinically relevant review articles. Emphasis was placed on evidence from United States and European cohorts, consistent with the clinical and epidemiologic focus of the review, although relevant studies from other settings were also considered when they provided important mechanistic or translational insights. Studies were selected according to their relevance, methodological robustness, clinical applicability, and contribution to one or more of the three thematic pillars of the review.

## 3. Immunopathogenesis and Contemporary Classification of Kidney Allograft Rejection

Kidney allograft rejection is not a single, discrete event but the clinical expression of a dynamic alloimmune process that starts at reperfusion and may persist for years. From the moment donor endothelium, tubular epithelium, and resident antigen-presenting cells are exposed to recipient blood, a coordinated network of innate and adaptive immune pathways is engaged. Direct, indirect, and semidirect allorecognition drive the activation of CD4+ and CD8+ T cells, B cells, plasma cells, and innate effector cells, including macrophages and natural killer (NK) cells, which converge on the kidney microvasculature and tubulointerstitial compartment to produce distinct but often overlapping patterns of tissue injury. Over time, these processes may evolve from active inflammatory lesions to chronic microvascular remodeling, transplant glomerulopathy, interstitial fibrosis, and tubular atrophy. Rejection is therefore better understood as a mechanistically heterogeneous continuum than as a series of isolated histologic snapshots [41,42,43,44,45,46,47,48,49].

### 3.1. Overview of Alloimmune Responses in Kidney Transplantation

From an immunologic perspective, KT creates a sustained confrontation between donor tissue and recipient immunity. The kidney allograft introduces a large burden of non-self-antigens, most prominently donor HLA molecules expressed on endothelial and tubular cells, into a recipient whose immune system is primed to recognize and eliminate such foreign signals. The resulting alloimmune response can be broadly separated into cellular and humoral components, but these are biologically interconnected rather than independent [50,51,52,53].

T-cell activation remains central to the initiation and amplification of allograft injury. Through direct, indirect, and semidirect pathways of allorecognition, recipient T cells recognize donor antigen, expand clonally, and acquire effector functions that promote interstitial inflammation, tubulitis, and, in some cases, vascular injury. In parallel, B cells and plasma cells generate DSAs, most commonly directed against HLAs, which bind the graft microvasculature and trigger endothelial injury through complement-dependent and complement-independent mechanisms. Fc receptor engagement on NK cells, monocytes, and other innate effectors further amplifies microvascular inflammation, including in cases in which classical complement staining is weak or absent [8,12,52,54,55,56,57,58].

These pathways do not operate in isolation. Ischemia–reperfusion injury (IRI) and surgical trauma release danger-associated molecular patterns that activate innate immunity, enhance antigen presentation, and lower the threshold for subsequent adaptive alloimmune responses. Recurrent or undertreated TCMR may facilitate DSA formation by increasing antigen presentation and local costimulatory signaling, whereas persistent humoral injury sustains endothelial activation, leukocyte recruitment, and profibrotic remodeling. The biological consequence is a self-reinforcing circuit in which inflammation, endothelial dysfunction, and maladaptive repair promote progression from active rejection to chronic injury [54,59,60,61,62].

Within this framework, the contemporary classification of kidney allograft rejection attempts to map underlying mechanisms onto clinicopathologic categories by linking histologic lesions, serologic findings, and, increasingly, molecular evidence. TCMR reflects predominantly tubulointerstitial cellular injury; ABMR reflects mainly microvascular endothelial injury driven by alloantibodies; and mixed or DSA-negative phenotypes occupy biologically and diagnostically intermediate zones. These categories remain clinically useful, but they should be interpreted as approximations of underlying biology rather than as fully discrete entities [16,18,63,64].

This mechanistic layering is also important for how rejection is later measured. Predominantly tubular and interstitial T-cell recruitment is more likely to generate inflammatory signals that map onto chemokine- and transcript-based readouts, whereas microvascular endothelial injury caused by DSAs, complement activation, and Fc receptor–dependent innate effectors is more likely to produce combined patterns of endothelial transcripts, glomerular/peritubular capillary lesions, and injury-release signals such as dd-cfDNA. Markers of net immunosuppression, such as TTV, sit even further upstream, because they do not measure rejection itself but the host environment in which rejection becomes more or less likely. In this sense, biomarkers are not competing labels for the same event: they are partial readouts of different biological levels of the same alloimmune process [52,53,54,55,56,57,58,59,62].

### 3.2. T Cell–Mediated Rejection (TCMR): Acute and Chronic

TCMR represents the classical cellular arm of alloimmune injury in kidney transplantation. It arises when donor alloantigens are recognized by recipient T cells through direct, indirect, and semidirect pathways, leading to clonal expansion of alloreactive CD4+ and CD8+ lymphocytes and recruitment of additional inflammatory cells into the interstitium and tubular epithelium. Activated T cells release cytokines such as IL-2, IFN-gamma, and TNF-alpha and induce cytotoxic injury through perforin- and granzyme-mediated pathways, thereby producing the characteristic pattern of tubulointerstitial injury that defines TCMR histologically [44,65,66,67,68,69,70,71].

Within the Banff framework, acute TCMR is defined by interstitial inflammation and tubulitis, with or without intimal arteritis. Banff 2019 retained the requirement for moderate interstitial inflammation involving more than 25% of nonscarred cortex together with tubulitis for grade IA/IB lesions, whereas higher grades require vascular involvement. In contemporary North American and European cohorts, biopsy-proven acute TCMR has become less frequent than in earlier eras, largely because of more effective induction therapy, therapeutic drug monitoring, and improved maintenance immunosuppression. Nevertheless, it remains clinically important, particularly in sensitized recipients, younger adults, and patients with underexposure to calcineurin inhibitors or nonadherence [7,16,18,72,73,74,75,76].

From a biological standpoint, however, TCMR should not be regarded as a uniform entity. Some episodes represent brisk, treatment-responsive cellular alloimmunity, whereas others occur in the context of chronic parenchymal injury, mixed rejection, or persistent low-grade inflammation within scarred tissue. The borderline category illustrates this complexity. Although borderline changes were historically considered of uncertain significance, more recent data suggest that persistent or late borderline lesions, especially in patients with DSAs or other evidence of alloimmune activation, may herald subsequent overt rejection and faster chronic injury accumulation. This supports a more contextual interpretation of borderline TCMR rather than a purely threshold-based one [8,12,24,77,78,79,80,81].

Chronic active TCMR further highlights the limitations of a simplistic acute-versus-chronic dichotomy. In these biopsies, active inflammation and tubulitis are superimposed on established interstitial fibrosis and tubular atrophy, indicating that cellular alloimmunity can remain operative even after chronic structural damage has become evident. Longitudinal studies suggest that recurrent, persistent, or subclinical TCMR contributes to progressive functional decline and is particularly harmful when accompanied by DSAs, microvascular inflammation, or transplant glomerulopathy [12,21,80,81,82].

Molecular studies have added further nuance. Not all biopsies that fulfill histological criteria for TCMR appear to represent the same degree of active alloimmune injury. Some show strong T-cell and IFN-gamma–related transcriptional signatures, whereas others display weaker or more heterogeneous molecular activity, raising the possibility that at least part of what is labeled histologic TCMR may reflect resolving injury or nonspecific inflammation rather than fully active rejection. This heterogeneity helps explain why the prognostic significance of TCMR is not identical across all cases. Clinically, isolated early acute TCMR often remains treatment responsive, whereas late, recurrent, or molecularly active TCMR is much more strongly linked to chronic scarring, de novo DSA formation, and transition toward mixed or humoral phenotypes. TCMR, therefore, remains highly relevant in the modern era, not only as an acute lesion but also as a contributor to long-term graft remodeling [18,24,83,84,85,86,87,88].

### 3.3. Antibody-Mediated Rejection (ABMR): Acute and Chronic Active

ABMR is the humoral expression of alloimmunity and is centered on endothelial injury within the graft microvasculature. In their canonical form, circulating DSAs bind donor antigens on glomerular and peritubular capillary endothelium, leading to complement activation, Fc receptor–mediated recruitment of innate effector cells, endothelial activation, and microvascular inflammation. Histologically, this is reflected by glomerulitis, peritubular capillaritis, and, in some cases, C4d deposition; over time, persistent endothelial injury promotes transplant glomerulopathy, basement membrane multilayering, capillary rarefaction, and chronic structural remodeling. Contemporary European and North American cohorts consistently identify caABMR as the dominant cause of death-censored graft loss in the modern era [8,89,90,91,92,93,94,95].

Mechanistically, ABMR is more than the mere presence of antibodies. The central lesion is sustained endothelial stress and injury within the graft’s microvasculature. Anti-HLA DSAs remain the main drivers, but non-HLA antibodies may contribute in selected patients, particularly when the histologic pattern is suggestive of humoral injury but conventional serology is incomplete or negative. Complement-dependent damage is important in many cases, but complement-independent pathways also matter, especially through NK-cell and monocyte activation via Fc receptors. This is particularly relevant for understanding C4d-negative ABMR, in which biologically active microvascular injury may persist despite the absence of a classical complement footprint [17,51,57,89,96,97,98,99,100,101,102,103,104,105].

The distinction between acute ABMR and chronic active ABMR is clinically useful but biologically porous. Acute ABMR captures active microvascular injury, often in the early post-transplant setting or in highly sensitized recipients. caABMR, by contrast, reflects the coexistence of ongoing activity with established chronic remodeling, especially transplant glomerulopathy. Rather than representing two unrelated states, these entities are better interpreted as points along a continuum from antibody-mediated endothelial activation to chronic microvascular remodeling and fibrosis [17,51,57,89,96,97,98,99,100,101,102,103,104,105].

DSA-negative ABMR-like phenotypes further illustrate this complexity. A subset of biopsies displays convincing histologic and/or molecular features of ABMR without detectable circulating DSAs on standard solid-phase assays. Possible explanations include assay limitations, transient or compartmentalized antibody responses, or non-HLA antibodies. Importantly, these cases often behave biologically and prognostically like conventional ABMR, suggesting that absence of detectable circulating DSAs does not exclude active antibody-mediated injury. The role of non-HLA antibodies remains clinically relevant but still incompletely defined. Antibodies directed against targets such as angiotensin II receptor type 1, endothelin A receptor, ARHGDIB, and other endothelial antigens have been associated with ABMR-like phenotypes or adverse outcomes in some cohorts, but findings remain heterogeneous because of assay variability, inconsistent cut-offs, and differences in phenotyping. For now, their interpretation should remain contextual and should not supersede convergent histologic, serologic, and molecular evidence [17,89,96,97,98,105].

### 3.4. Mixed Rejection and DSA-Negative Phenotypes

A substantial proportion of allograft biopsies do not fit neatly into “pure” TCMR or “pure” ABMR. Instead, they display overlapping tubulointerstitial and microvascular lesions, often in association with DSAs and variable C4d staining. Mechanistically, this overlap is expected rather than exceptional. T-cell–driven inflammation can enhance endothelial activation, cytokine production, and germinal center responses, thereby facilitating humoral alloimmunity, whereas persistent antibody-mediated injury sustains a proinflammatory microenvironment that promotes continued T-cell recruitment and tissue remodeling. In longitudinal series, patients rarely transition abruptly from an isolated TCMR episode to “pure” caABMR; instead, many traverse a mixed phase in which cellular and humoral mechanisms coexist and feed into each other. Mixed rejection should therefore be understood less as the accidental coexistence of two separate entities than as the integrated expression of converging alloimmune pathways. Table 1 provides a concise comparison of TCMR, ABMR, and mixed rejection, emphasizing both their distinguishing features and their biological overlap [7,8,12,21,106,107].

This interpretation is clinically important because mixed phenotypes are consistently associated with worse outcomes than isolated TCMR or isolated ABMR. Several cohort studies have shown that patients whose biopsies demonstrate both significant interstitial/tubular inflammation and microvascular inflammation have higher rates of de novo or persistent DSAs, faster eGFR decline, more rapid development of transplant glomerulopathy, and higher death-censored graft loss than those with isolated TCMR or isolated ABMR. Molecular profiling supports this biology, as mixed lesions often harbor concurrent T-cell–associated and endothelial/ABMR-associated transcripts, suggesting that both arms of alloimmunity remain active [7,8,18,84,107].

DSA-negative phenotypes represent a related gray zone. A meaningful minority of biopsies with ABMR-like histology or molecular signatures lack detectable anti-HLA DSAs on single-antigen bead assays. These cases expose an important limitation of current classification systems: static serologic testing may not fully capture the breadth, timing, or compartmentalization of humoral alloimmunity. This is why Banff 2019 and especially Banff 2022 have moved toward accepting validated molecular evidence of antibody–endothelium interaction in selected, carefully adjudicated cases. Together, mixed and DSA-negative phenotypes act as stress tests for the current classificatory framework, showing where morphology and serology remain informative but incomplete [16,17,18,22,96].

In this context, the dominant cellular process also helps explain which diagnostic signals are more likely to be detected. Predominantly tubulointerstitial T-cell recruitment is more likely to align with chemokine- and T-cell transcript–enriched readouts, whereas antibody-driven endothelial injury with complement activation and Fc receptor–dependent innate effector recruitment is more likely to generate microvascular lesions, endothelial molecular programs, and injury-release signals such as dd-cfDNA. Mixed rejection is more difficult precisely because it may produce composite or shifting signatures across these layers. This is one reason why no single biomarker can reliably adjudicate phenotype in isolation, particularly once chronic injury, infection, or treatment effects begin to overlap with active alloimmunity [7,8,12,16,17,18,22,96].

### 3.5. Banff 2019 and 2022: Where Are We Now?

Three decades after its inception, the Banff classification remains the global reference standard for kidney allograft pathology, and the 2019 and 2022 updates represent important milestones in its evolution. Banff 2019 clarified the criteria for chronic active TCMR, narrowed the borderline category to reduce overdiagnosis, and reaffirmed the triad required for ABMR diagnosis: histologic evidence of microvascular injury, evidence of current or recent antibody–endothelium interaction, and serologic DSAs or validated substitutes. Banff 2022 further reappraised microvascular inflammation thresholds and formally recognized biopsy transcriptomics as an additional line of evidence, particularly useful in C4d-negative or DSA-negative cases [16,17,18,91,92,104,108,109].

These changes strengthened the diagnostic framework, but they also highlighted its remaining limits. Interobserver variability remains relevant for several key lesions, especially tubulitis, interstitial inflammation, and microvascular inflammation, and the resulting diagnostic complexity is not always matched by equally precise therapeutic pathways [38,39]. Mixed rejection, DSA-negative ABMR, late borderline lesions, and inflammation in scarred parenchyma continue to challenge diagnostic consistency and clinicopathologic interpretation. Moreover, the classification remains largely biopsy-centric and episodic, capturing tissue injury at single time points rather than fully integrating longitudinal serologic, molecular, and clinical information. Banff therefore remains indispensable, but increasingly insufficient on its own. This is precisely why a rejection-focused precision framework cannot rely on morphology alone: once the biologic heterogeneity of rejection is acknowledged, the central question becomes which additional signals best capture injury, inflammation, immune context, and molecular phenotype at each stage of the process [16,17,18].

## 4. Epidemiology and Clinical Impact of Rejection in the Modern Era

Despite major gains in early post-transplant outcomes, the burden of kidney allograft failure is increasingly concentrated in the late post-transplant period and is largely driven by alloimmune injury. Contemporary registry analyses from the United States and Europe show excellent 1-year graft survival, but only incremental improvement in long-term outcomes, underscoring that the main unmet need now lies beyond the first post-transplant year. In this setting, chronic rejection, particularly caABMR, has emerged as a leading cause of death-censored graft loss, shifting clinical attention from early overt rejection to persistent and often indolent forms of alloimmune injury [2,3,7,8,9,110,111,112,113].

### 4.1. Incidence of Acute and Chronic Rejection in Contemporary Cohorts

In the current era, biopsy-proven acute rejection (BPAR) within 1 year generally occurs in approximately 5–12% of recipients across large contemporary US cohorts, with similar estimates reported in European practice, although rates remain higher in immunologically high-risk patients. Subclinical rejection detected on surveillance biopsies remains variable, largely because of differences in biopsy protocols and maintenance immunosuppression. For ABMR, pooled contemporary estimates suggest a wide incidence range, reflecting differences in sensitization burden, donor type, immunologic compatibility, and follow-up duration. Longitudinally, the burden of rejection increasingly shifts toward humoral injury, with cumulative ABMR rising over time while TCMR becomes less frequent in long-term follow-up. This temporal shift is clinically important because it aligns with the pattern of late graft loss seen in modern transplant practice [114,115,116,117,118,119].

Across contemporary cohorts, rejection is also the leading proximate cause of death-censored graft loss, with caABMR accounting for the largest share of late alloimmune failure. These data reinforce a central point of this review: the major challenge in modern kidney transplantation is no longer the prevention of early rejection alone, but the earlier detection and better characterization of persistent alloimmune injury. Table 2 summarizes key epidemiologic figures for acute and chronic rejection across contemporary US and European cohorts, contextualizing the burden outlined in Section 3 [7,9,110,120,121].

### 4.2. Risk Factors and Vulnerable Populations

Rejection risk reflects the interaction of immunologic compatibility, immunosuppressive exposure, peri-operative injury, and recipient vulnerability. Molecular HLA mismatches, particularly at the DR and DQ loci, remain among the strongest predictors of de novo DSA formation and subsequent ABMR. Preformed DSAs, early post-transplant DSA detection, and persistent rather than transient DSAs are also associated with higher rates of ABMR and inferior graft outcomes. At the population level, the continued presence of highly sensitized candidates on transplant waiting lists underscores the ongoing relevance of this risk domain [99,122,123,124,125,126,127].

Immunosuppressive exposure is another major determinant of rejection. Tacrolimus intrapatient variability and time outside the target range are consistently associated with dnDSA development, rejection, and poorer graft outcomes. Pharmacogenetic variability, including CYP3A5 expression, may further contribute to early underexposure and higher rejection risk in selected populations. These observations support closer attention to individualized drug exposure rather than reliance on nominal dosing alone [128,129,130,131,132].

Delayed graft function (DGF) also remains a clinically relevant amplifier of alloimmune risk. In addition, certain recipient groups appear particularly vulnerable, including adolescents and young adults, in whom rejection risk is shaped by both biology and adherence, and some ethnically diverse populations, in whom long-term outcome disparities likely reflect a combination of immunologic, pharmacogenomic, and structural determinants. Non-HLA antibody burden may add further risk in selected settings, although interpretation remains limited by assay heterogeneity and incomplete standardization. Collectively, these data support a risk-adapted approach to surveillance and follow-up, rather than uniform post-transplant monitoring for all recipients [133,134,135,136,137,138].

### 4.3. Donor and Organ Quality as Modifiers of Rejection Risk

Donor and organ quality are not classic immunologic risk factors, but they strongly shape the injury environment in which alloimmunity develops. Variables such as donor age, KDPI/KDRI, expanded-criteria donor status, and procurement pathway influence ischemia–reperfusion injury, delayed graft function, and long-term graft reserve. Among these, DGF is the clearest bridge between organ quality and rejection risk, being consistently associated with higher rates of acute rejection, inferior early function, and worse graft survival [133,135,136,138,139,140,141,142,143,144,145,146].

Donation after circulatory death and donation after brain death represent biologically distinct injury trajectories, with differing patterns of warm ischemia, endothelial stress, and inflammatory priming. These differences are relevant because they may modulate the threshold at which alloimmune injury becomes clinically manifest. Emerging metabolomic and spectral approaches suggest that more refined biological phenotyping of donor organs may eventually complement conventional donor scores, but these strategies remain preliminary and are not yet ready for routine rejection risk stratification [140,146,147,148,149,150].

Taken together, the epidemiology of rejection in the modern era supports a clinically important shift: risk is increasingly shaped by the interaction of baseline immunologic incompatibility, ongoing drug exposure, and the degree of early graft injury. This reinforces the need for more selective and biologically informed surveillance strategies, especially in patients at higher risk of late alloimmune injury.

## 5. Conventional Diagnostic Framework for Kidney Allograft Rejection

The conventional framework for kidney allograft rejection still rests on three pillars: functional markers of graft injury, serological evidence of alloimmunity, and the allograft biopsy as the reference standard. Each component, however, has important limitations, particularly in chronic, subclinical, or biologically heterogeneous rejection.

### 5.1. Functional Markers: Creatinine, eGFR, Proteinuria

In day-to-day practice, most centers still screen for rejection using serum creatinine, eGFR, and proteinuria because these tests are ubiquitous, inexpensive, and longitudinally available. However, their diagnostic performance for active alloimmune injury is modest.

Serum creatinine is a lagging indicator of graft injury. A measurable rise may occur only after substantial nephron loss has already taken place, and creatinine kinetics are influenced by muscle mass, hydration status, and drug-related effects. Contemporary reviews consistently emphasize that creatinine and eGFR have low sensitivity for subclinical or early rejection and limited specificity for distinguishing TCMR from ABMR or from non-immune causes of dysfunction, such as calcineurin inhibitor toxicity, ischemic injury, or recurrent disease. This helps explain why many cases of caABMR present with deceptively stable kidney function until chronic injury is already established [151,152].

Proteinuria is also clinically relevant, but it is not specific for rejection. It often reflects glomerular damage and correlates with adverse long-term outcomes, yet it may increase in recurrent or de novo glomerulonephritis, hemodynamic injury, or medication-related toxicity. Taken together, functional markers remain necessary for context and triage, but they are poor stand-alone tools for early or subtype-specific rejection diagnosis [151,153].

### 5.2. Donor-Specific Antibodies (DSAs) and Complement-Binding Assays

The detection and characterization of anti-HLA DSAs are central to identifying humoral alloimmunity and stratifying risk for ABMR and graft loss. Single-antigen bead (SAB) assays provide semi-quantitative measures of IgG binding to HLA epitopes, and robust evidence links both preformed and de novo DSAs to active and chronic ABMR, transplant glomerulopathy, and inferior death-censored graft survival. At the same time, DSAs must be interpreted as biologically important but not self-sufficient signals. Their presence indicates alloimmune risk, but not all DSAs are equally pathogenic, and their absence does not exclude ABMR [16,17,154,155,156,157,158,159].

This interpretation is limited by several well-recognized technical issues. SAB mean fluorescence intensity is not a linear measure of antibody burden and may be influenced by the prozone effect, antigen density, and epitope sharing. Dilution and EDTA treatment may be necessary to reveal true signal in high-titer sera, and interlaboratory variation in assay thresholds continues to affect clinical interpretation. These limitations are especially relevant in cases of DSA-negative ABMR-like injury, where histology or molecular diagnostics may indicate active antibody-mediated damage despite negative circulating serology [17,160,161,162].

Complement-binding assays such as C1q and C3d have been explored as a way to distinguish more injurious DSAs from less pathogenic antibodies. Several studies suggest that complement-binding DSAs are associated with more severe injury and worse graft outcomes than non-binding antibodies. Even so, these assays do not replace biopsy and should not be interpreted in isolation. Their value lies in refining risk stratification when considered together with DSA class, strength, persistence over time, histology, and other adjunctive markers [17,163,164].

### 5.3. The Allograft Biopsy: Strengths and Limitations

The kidney allograft biopsy remains the reference standard for diagnosing and grading rejection because it directly establishes the presence and pattern of tissue injury. It defines the lesions required for Banff-based classification, including interstitial inflammation, tubulitis, intimal arteritis, microvascular inflammation, transplant glomerulopathy, and ancillary findings such as C4d deposition and chronicity indices. It also provides the tissue substrate for biopsy-based molecular diagnostics, including transcriptomic approaches used to clarify ambiguous cases or support ABMR diagnosis when conventional histology and serology are inconclusive [16,17,18,63,73,83,84].

Its limitations, however, are equally important. Sampling variability remains a persistent problem, especially for patchy lesions such as borderline inflammation and focal microvascular injury. Interobserver variability also affects reproducibility, particularly for intermediate grades of tubulitis, interstitial inflammation, and capillaritis. Biopsy is invasive, with a low but real risk of bleeding and other complications, even in experienced centers. In addition, histology and molecular readouts do not always align perfectly, reminding us that even tissue-based diagnosis requires contextual interpretation [78,165,166,167,168,169].

Biopsy, therefore, remains indispensable, but it is not infallible. Its main strength is phenotype definition; its main weaknesses are invasiveness, sampling error, and limited capacity to capture rejection as a longitudinal process rather than a single time-point event.

### 5.4. Surveillance vs. For-Cause Biopsy Strategies

The role of protocol biopsy remains debated, and practice continues to vary across transplant programs in North America and Europe. In some centers, surveillance biopsies are used to detect subclinical cellular or humoral rejection and to capture evolving chronic injury before overt graft dysfunction becomes apparent. In others, concerns related to procedural risk, cost, logistics, and uncertain impact on long-term outcomes limit their routine use. Even with modern ultrasound-guided techniques, protocol biopsies still impose a procedural burden, although major complications remain uncommon in experienced centers [117,166,170,171,172].

For-cause biopsy, therefore, remains the standard approach when there is a rise in serum creatinine, new or worsening proteinuria, emergence or strengthening of DSAs, or another signal suggesting active graft injury. More recently, non-invasive biomarkers such as dd-cfDNA have been proposed as adjuncts to improve patient selection for biopsy and to increase the diagnostic yield of tissue evaluation. This has supported a more selective, risk-adapted strategy in which protocol biopsies may be reserved for higher-risk settings, while biomarker-guided triage helps identify which patients are most likely to benefit from tissue sampling [17,24,32,87,168,173].

Together, functional markers, DSA testing, and biopsy remain the foundation of conventional rejection assessment, but each has important limitations that constrain early and precise diagnosis. Table 3 summarizes the main strengths and limitations of these conventional diagnostic tools.

The main limitation of the conventional framework is therefore not lack of relevance, but lack of precision.

## 6. Emerging Biomarkers and Molecular Diagnostics of Rejection

Despite the continued central role of serum creatinine/eGFR, DSAs, and the biopsy, the last five years have expanded the range of tools available to detect and characterize kidney allograft rejection. These emerging approaches include dd-cfDNA, urinary chemokines such as CXCL9 and CXCL10, blood gene-expression profiling (GEP), Torque Teno Virus (TTV) load, proteomic and metabolomic signatures, and biopsy-based transcriptomics. Their clinical appeal lies in the possibility of identifying alloimmune injury earlier, more dynamically, and sometimes less invasively than conventional tools allow [17,24,27,174].

At the same time, this expanding biomarker landscape requires a more structured interpretive logic. In practical terms, rejection biomarkers can be grouped into four biologically distinct classes: (i) injury-release signals, which indicate that tissue damage is occurring but do not define its cause; (ii) inflammatory readouts, which reflect intragraft immune activation but may not distinguish alloimmune from infectious or nonspecific inflammatory states; (iii) markers of net immunosuppression, which help explain the host context in which rejection becomes more or less likely without defining phenotype; and (iv) tissue-level molecular classifiers, which come closest to phenotype adjudication but still require clinicopathologic correlation. This distinction matters because the most common interpretive error in precision medicine is not the absence of signal, but the misassignment of meaning: using an injury signal as if it were mechanism-specific or treating an inflammatory signal as if it established diagnosis on its own. The value of emerging biomarkers, therefore, lies less in isolated performance than in whether each test is used at the correct biological level and clinical decision point [24,32]. In practical terms, three recurrent errors follow from this framework: reading injury as if it were mechanism-specific, reading inflammation as if it established diagnosis on its own, and reading immune context as if it defined phenotype.

Table 4 summarizes the principal emerging biomarkers discussed in this review, highlighting what each measures biologically, its main strengths and limitations, and its current potential role in rejection assessment.

### 6.1. Donor-Derived Cell-Free DNA (dd-cfDNA)

dd-cfDNA is one of the most intensively studied non-invasive biomarkers in KT. It quantifies the fraction of circulating cell-free DNA derived from the allograft and is therefore best understood as a marker of graft injury rather than a direct marker of a specific rejection mechanism. In practice, an increased donor-derived fraction may accompany active rejection, but it may also rise in other forms of graft injury, including IRI, BK virus nephropathy, or a recent biopsy. Its biological value lies in signaling that the graft is being injured; its main limitation is that it does not, by itself, explain why [25,30,31,32,33].

Recent meta-analyses in kidney transplantation have shown that elevated dd-cfDNA is associated with both TCMR and ABMR, with pooled diagnostic performance that exceeds that of conventional functional markers in many settings. A large multicenter transatlantic observational study further showed that dd-cfDNA adds incremental diagnostic value beyond standard-of-care monitoring for active rejection. These findings support its role as a useful adjunct in both surveillance and for-cause evaluation, particularly when interpreted together with DSAs, histology, and clinical data [30,31,32].

However, dd-cfDNA should not be presented as a mechanistically specific biomarker of rejection. It is better viewed as a sensitive injury-release signal than as a definitive discriminator of alloimmune phenotype. Its performance varies according to assay platform, threshold selection, timing after transplantation, and the presence of competing causes of graft injury. This is especially important when discussing precision medicine: dd-cfDNA may improve detection and triage, but it does not by itself distinguish causal alloimmune pathways from downstream tissue damage [25,33].

Clinically, dd-cfDNA appears most useful when used serially and relationally rather than as a stand-alone binary test. A rising value in a DSA-positive recipient or in a patient with otherwise unexplained graft dysfunction should lower the threshold for tissue evaluation, whereas a persistently low value in a clinically stable patient may help defer an immediate biopsy in selected contexts [32,33]. Its greatest strength is probably not the replacement of biopsy but better selection of who most needs biopsy and when. Table 5 summarizes the main contemporary studies evaluating dd-cfDNA across different cohorts, assay platforms, and clinical settings, highlighting both its diagnostic potential and the methodological heterogeneity that still affects interpretation.

### 6.2. Urinary Chemokines CXCL9 and CXCL10

Urinary CXCL9 and CXCL10 are among the most biologically appealing biomarkers of rejection because they reflect interferon-gamma–driven intragraft inflammation. Produced largely by tubular epithelial and endothelial cells in response to inflammatory signaling, they provide a non-invasive readout of the alloimmune microenvironment and may become abnormal before overt changes in serum creatinine or eGFR. Compared with dd-cfDNA, these chemokines are closer to an inflammatory pathway readout; however, they still do not provide absolute specificity for rejection [26,27,28,29].

Recent studies suggest that urinary CXCL9 and CXCL10 can help detect active rejection, including subclinical rejection, and may offer high negative predictive value in selected clinical settings. Their value appears greatest as rule-out tools and as adjuncts in biopsy triage, particularly when combined with DSAs, graft function, and other biomarkers. More recent implementation-focused studies have also clarified important pre-analytical and analytical issues, including specimen handling, creatinine normalization, and assay standardization [26,27,28,29].

Still, these markers must be interpreted cautiously. Elevated urinary chemokines are not specific to alloimmune rejection and may also be seen in urinary tract infection, BK virus infection, or other inflammatory states affecting the graft. They are therefore better understood as markers of intragraft immune activation than as definitive markers of a specific rejection phenotype. This is precisely the type of distinction that matters in a precision-medicine framework: urinary CXCL9/CXCL10 may indicate that inflammation is present, but they do not independently determine whether the dominant driver is T-cell–mediated, antibody-mediated, infectious, or mixed [27,29].

For clinical use, urinary chemokines seem most informative when integrated into a layered diagnostic strategy rather than interpreted in isolation. In patients with stable kidney function, they may help identify subclinical inflammatory activity; in patients with graft dysfunction, they may help prioritize biopsy when combined with dd-cfDNA, DSAs, and clinical context [26,29,178,179].

Table 6 summarizes the principal studies on urinary CXCL9 and CXCL10, illustrating both their value as non-invasive markers of intragraft inflammation and the practical limitations that currently prevent them from being interpreted as stand-alone diagnostic tools.

Their main strength lies in non-invasive inflammatory surveillance; their main limitation lies in imperfect specificity. Beyond dd-cfDNA and urinary chemokines, however, a broader group of blood- and urine-based biomarkers is emerging, each capturing a different layer of rejection biology or of the host response to immunosuppression.

### 6.3. Other Urine- and Blood-Based Biomarkers

Beyond dd-cfDNA and urinary chemokines, the current biomarker landscape includes a broader group of tools that capture distinct but complementary layers of biology. These include blood GEP as a readout of systemic immune activation, TTV load as a surrogate of net immunosuppression, proteomic and peptidomic panels that identify candidate protein signatures of graft injury, extracellular vesicle and exosome-derived signatures that provide cell-of-origin–enriched molecular cargo, and label-free spectral fingerprints such as Fourier-transform infrared (FTIR) spectroscopy that summarize global biochemical perturbation. The common principle is orthogonality: these approaches do not measure the same biological process and should not be interpreted as interchangeable.

Blood gene-expression profiling. GEP panels, typically based on qPCR, quantify immune programs enriched for T-cell and interferon-gamma axis activity. Their main attraction is that they move closer to immune activation than injury-release markers alone. In a real-life, multi-site appraisal with embedded validation, the combination of GEP and dd-cfDNA outperformed either assay alone for identifying patients most likely to require biopsy, including cases of subclinical activity that might otherwise be missed by creatinine/eGFR and static serology [181]. This supports a layered strategy in which immune-activation and injury-release signals are paired rather than used in isolation. At the same time, GEP does not directly resolve tissue phenotype or etiology and remains best viewed as an adjunctive triage tool rather than a replacement for biopsy.

Torque teno virus load. TTV DNAemia is conceptually different from rejection biomarkers because it acts primarily as a gauge of net immunosuppression rather than as a direct rejection test. A 2023 systematic review and meta-analysis concluded that TTV offers useful risk stratification but only modest discrimination for acute rejection, and therefore should not be used as a diagnostic substitute for dd-cfDNA, urinary chemokines, or biopsy [182]. More recent studies and perspectives emphasize that TTV is best interpreted longitudinally, alongside tacrolimus exposure and variability, to help balance the competing risks of under- and over-immunosuppression [174,183,184,185]. In other words, TTV may help contextualize rejection risk, but it does not itself establish rejection phenotype.

Proteomic and peptidomic panels. High-resolution mass spectrometry platforms are generating increasingly promising candidate panels in both serum and urine. A 2024 study identified a three-protein serum signature (SAA1, AHSG, IGFBP2) that classified acute rejection with internal validation, while a multicenter European study applied urinary micro-LC–TOF-MS to profile graft injury phenotypes and explicitly examined demographic and treatment-related confounders. These approaches are attractive because they may capture more specific molecular consequences of injury than global functional markers. However, most proteomic panels remain in discovery or early validation phases, and assay harmonization, external validation, and phenotype anchoring remain essential before clinical adoption [87,186,187,188].

Extracellular vesicles and exosome-derived signatures. EVs, particularly exosomes released by tubular epithelial cells, endothelial cells, and immune cells, offer a biologically appealing platform because they carry proteins, lipids, mRNA, microRNA, and cell-free DNA in a protected form and may be enriched for kidney-derived material when measured in urine. Proof-of-concept and early validation studies suggest that quantitative and qualitative changes in urinary EVs may discriminate against acute rejection from stable graft function and from alternative causes of dysfunction and may also reflect chronic injury. Even so, the translational hurdles are substantial: pre-analytical handling, heterogeneity in isolation platforms, and lack of standardized normalization still limit comparability across studies. For now, EV-derived signatures should be regarded as high-potential but still investigational biomarkers [168,189,190,191,192].

Label-free spectral fingerprints (FTIR) paired with machine learning. FTIR-based approaches differ from the biomarkers above in that they do not quantify a single molecular analyte; instead, they generate a global biochemical fingerprint reflecting proteins, lipids, nucleic acids, and glycoconjugates. In kidney transplantation, recent studies benchmarked against biopsy and analyzed with supervised machine learning have reported very high apparent accuracy for distinguishing rejection from non-rejection and, in some settings, for discriminating TCMR from ABMR. The attraction of FTIR lies in its speed, low sample requirement, low per-test cost, and potential role as a front-end triage tool. However, these findings still require rigorous multicenter standardization, confounder control, and demonstration of added value beyond current standards such as dd-cfDNA, urinary CXCL9/CXCL10, and DSAs. At present, FTIR should, therefore, be viewed as promising but still investigational [78,167,193].

Taken together, these additional biomarkers expand the biological depth of rejection assessment, but they also illustrate a central challenge of precision medicine: more signals do not automatically mean more specificity. Their interpretability depends on where they sit along the causal chain of rejection. Injury-release markers such as dd-cfDNA are most sensitive when active tissue damage is already occurring, but they are not inherently specific for alloimmunity and may also rise with ischemia–reperfusion injury, BK virus nephropathy, or recent biopsy. Inflammatory readouts such as urinary CXCL9/CXCL10 are biologically closer to intragraft immune activation, yet they may also increase in infection or other inflammatory states and therefore cannot, by themselves, distinguish TCMR, ABMR, mixed rejection, or non-alloimmune injury. Markers of net immunosuppression such as TTV provide a different layer of information altogether: they may help explain why rejection risk is rising or falling, but they do not establish rejection phenotype. Proteomic, extracellular-vesicle, and FTIR-based approaches may add complementary molecular depth, but at present they remain especially vulnerable to overlap between causal mechanisms and downstream consequences of injury. In clinical practice, the most common interpretative error is therefore to treat a biologically informative signal as if it were a diagnosis. A rejection-focused biomarker strategy should instead ask a more disciplined question: does this test indicate active tissue injury, intragraft inflammation, inadequate immunosuppression, or a molecular phenotype that still requires histological adjudication? Table 7 summarizes representative studies of these additional blood- and urine-based biomarkers and highlights both their current stage of validation and the interpretive limits that must be considered in practice.

### 6.4. Molecular Microscope and Gene Expression Profiling of Biopsies

Conventional histology remains central to rejection diagnosis, but it is limited by sampling variability, interpretive subjectivity, and difficulty in resolving borderline inflammation, mixed phenotypes, or ABMR-like injury when C4d staining or circulating DSAs are absent or equivocal. Biopsy-based transcriptomics, including the molecular microscope approach (MMDx), adds an orthogonal signal by profiling coordinated T-cell and endothelial/antibody-mediated programs directly within the allograft. In this sense, it differs from circulating biomarkers: rather than signaling injury or inflammation indirectly, it interrogates tissue-level molecular activity itself. The Banff 2022 Kidney Meeting Report formally acknowledged validated biopsy transcript signatures as alternative evidence of antibody–endothelium interaction in selected cases, particularly when conventional serology or C4d staining is inconclusive [17,195].

Recent studies suggest that tissue transcriptomics may improve phenotype adjudication when histology and serology are discordant. A contemporary review of the MMDx methodology summarized how microarray- and RNA-seq–based classifiers align molecular archetypes with rejection phenotypes and outcomes, while a more recent real-world analysis showed that continuous molecular scores can identify subthreshold rejection activity and track response or relapse beyond morphology alone. These features are attractive because they move the field from binary labels toward graded molecular activity, which may be useful both clinically and in therapeutic trials [20,195].

At the same time, MMDx should not be presented as a simple replacement for conventional pathology. A 10-center, 474-biopsy multicenter implementation study documented substantial variability in when biopsy transcriptomics is used, how results are interpreted, and how Banff 2022 histology is reconciled with MMDx sign-outs, underscoring the need for standardized indications, analytical pipelines, and interpretation thresholds before broader routine adoption. Likewise, disagreement between molecular and histologic calls should not automatically be viewed as error. Recent work has proposed placing tissue transcriptomics explicitly within test relationships, showing that when MMDx, histology, DSAs, and dd-cfDNA are considered within shared rejection classes, discordance may itself convey clinically useful information, particularly in DSA-negative or C4d-negative ABMR-like cases. In parallel, an integrated tissue-plus-plasma analysis showed that elevated dd-cfDNA corresponds to three distinct molecular states in the allograft: canonical ABMR, recent parenchymal injury, and TCMR, reinforcing the value of pairing circulating injury signals with tissue molecular programs [165,196,197].

These observations support a more balanced view of biopsy transcriptomics. Its main strengths are phenotype clarification when histology and serology are discordant, quantitative assessment of disease activity, and the potential to reduce part of the subjectivity inherent to purely histologic scoring. Its main limitations remain access, cost, turnaround time, intercenter variability, and the need for pre-specified rules on how to interpret discordant molecular and histologic results. In addition, MMDx does not solve every diagnostic problem. Single-center discordance analyses indicate that it may be less informative in some isolated tubulitis/arteritis patterns and does not replace full clinicopathologic correlation or the assessment of recurrent native disease. As a further strength, molecular profiling has also been used to interpret antirejection trials, with genome-wide biopsy analysis in treated ABMR mapping treatment response and relapse at the pathway level, thereby illustrating how molecular endpoints may complement histology and clinical laboratory data in interventional studies [17,20,165,169,196,198,199].

Taken together, these data suggest that biopsy transcriptomics adds real value when histology and serology are discordant, but its interpretation remains strongest when anchored to the broader clinicopathologic context. Table 8 summarizes representative studies of biopsy transcriptomics and MMDx in kidney allograft rejection, emphasizing both their diagnostic promise and the practical limitations that still affect implementation. Once these histologic, serologic, and molecular layers are generated, the next challenge is how to integrate them in a way that is interpretable, generalizable, and clinically useful. This is where artificial intelligence and machine learning may have an important role, particularly as tools for multimodal integration and decision support [20,165,169,195,196,197,198,199].

Viewed comparatively, these approaches do not solve the same diagnostic problem. dd-cfDNA currently has the strongest multicenter support for non-invasive injury triage, but its interpretability declines when graft injury is driven by infection, recent biopsy, or other non-alloimmune causes. Urinary CXCL9/CXCL10 are often better suited to ruling out active intragraft inflammation, yet their specificity falls in BK virus infection, urinary tract infection, and other inflammatory states, and threshold performance may vary across platforms and normalization strategies. GEP and TTV add contextual information rather than phenotype resolution and are therefore most useful when paired with injury or inflammatory markers rather than used as stand-alone surrogates of rejection. By contrast, biopsy transcriptomics offers the strongest phenotype adjudication once tissue is obtained, but its added value is constrained by cost, access, intercenter variability, and persistent discordance with conventional histology in some patterns. In practical terms, the main limitation of the field is no longer lack of signal, but the risk of applying a biologically informative test outside the context for which it is most interpretable.

## 7. Artificial Intelligence and Machine Learning in Kidney Allograft Rejection

AI and ML are increasingly being positioned as integrative tools across the kidney-transplant continuum, from donor–recipient matching and perioperative risk estimation to long-term surveillance for rejection and graft failure. Recent reviews emphasize that the value of these models should not be judged by accuracy alone, but by whether they improve clinical decision-making, reduce uncertainty, and fit within real-world transplant workflows. In the context of rejection-focused precision medicine, the main promise of AI is not to replace pathology, serology, or biomarkers, but to combine them more effectively [37,200,201,202].

### 7.1. Risk Prediction and Longitudinal Prognostication

On the risk-prediction side, ML models have moved beyond small proof-of-concept studies. Early work suggested that algorithms such as random forests and gradient boosting could outperform traditional regression when detailed donor and recipient data were available, including for early post-transplant rejection. More recently, a machine-learning–based nomogram built on six clinical and laboratory predictors improved long-term graft-survival prediction over conventional Cox models in a 20-year follow-up cohort, while a multi-step precision pathway for heterogeneous kidney transplant recipients further illustrated how sequential modeling may refine survival prediction in more complex populations. Time-to-event approaches such as random survival forests are particularly attractive in transplantation because they can model non-linear interactions and changing risk over time while still allowing some degree of interpretability through variable-importance ranking and partial-dependence analyses [200,203,204,205].

### 7.2. Digital Pathology and Automated Lesion Assessment

Digital pathology is one of the most clinically tangible AI applications in kidney allograft rejection. Banff-based diagnosis still depends on human interpretation of biopsy slides, a process known to suffer from interobserver variability, particularly for borderline inflammation, microvascular injury, and chronic lesions. Deep-learning models trained on whole-slide images have begun to classify rejection versus non-rejection and, in some studies, to further subtype rejection phenotypes with promising performance. Other pipelines have used instance segmentation or modular lesion-based analysis to quantify morphologic patterns such as interstitial fibrosis and tubular atrophy, thereby reducing some of the subjectivity inherent to manual scoring. Broader syntheses of ML in kidney allograft rejection increasingly favor modular, lesion-specific architectures over monolithic classifiers, particularly for Banff-aligned tasks such as glomerulitis, peritubular capillaritis, interstitial inflammation, and tubulitis [38,39,206,207,208].

Even so, digital pathology should not be framed as a replacement for expert renal pathology. Its more realistic near-term role is as a decision-support layer: prescreening slides, highlighting suspicious regions, standardizing lesion quantification, and potentially acting as a second reader in complex cases. This distinction is important for both scientific credibility and clinical adoption. Fully automated histopathologic diagnosis remains limited by annotation quality, center-specific staining and scanning variation, and the challenge of aligning image features with clinically meaningful endpoints rather than with local labeling habits alone [39,208].

### 7.3. Multimodal Integration: Biomarkers, Histology, and Clinical Data

The area in which AI may be most aligned with the concept of precision medicine is multimodal integration. Several strands of work are beginning to combine injury biomarkers, inflammatory biomarkers, histology, and clinical variables into more dynamic prediction frameworks. dd-cfDNA is a particularly obvious candidate for this approach. Large multicenter studies have already shown that dd-cfDNA adds diagnostic value beyond serum creatinine, proteinuria, and DSAs for detecting active rejection. More recent work demonstrated that combining fractional and absolute dd-cfDNA improves diagnostic performance for rejection, especially ABMR, compared with fractional dd-cfDNA alone, while prospective multicenter data linked dd-cfDNA dynamics after biopsy-proven rejection to subsequent graft loss and recurrent rejection [32,173,209,210].

Similar integrative logic is beginning to emerge for blood gene-expression profiling and urinary chemokines. Real-world cohorts have shown that combining blood GEP with dd-cfDNA improves non-invasive detection of both clinical and subclinical rejection compared with either test alone. In practice, this suggests that the real contribution of AI may not be the creation of a single superior biomarker, but rather the derivation of context-sensitive decision rules that combine dd-cfDNA trajectories, uCXCL9/CXCL10, DSA evolution, tacrolimus exposure, TTV load, prior biopsy findings, and longitudinal graft function. At the broader systems level, ML-driven approaches have also been explored for donor–recipient matching and kidney allocation, with recent reviews explicitly linking predictive performance to ethical and governance considerations [173,211].

### 7.4. Interpretability, Validation, and Clinical Implementation

This is also the point at which the main limitations of AI become impossible to ignore. Across transplantation, most AI models are still developed in single-center cohorts or retrospective registry datasets with variable outcome definitions, missing data, selection bias, and limited external validation. Apparent gains in discrimination may therefore fail to translate across centers, populations, or time. For rejection-focused applications, external validation is not a final technical step; it is the central barrier to clinical credibility [24,35,37,43,212]. These concerns are particularly relevant in pediatric recipients and in ethnically diverse populations, in whom biomarker performance, baseline immunologic risk, and the generalizability of AI models may differ from the adult populations on which most current studies are based.

Interpretability is equally critical. Many models remain “black boxes,” which reduces clinician trust, complicates regulatory pathways, and makes it harder to understand whether a model is learning biologically meaningful structure or merely reproducing confounded correlations. This is why recent work increasingly includes more interpretable outputs, including nomograms, partial-dependence plots, variable-importance rankings, and Shapley-value–style visualizations. In the transplant setting, AI is far more likely to be adopted as a decision-support tool than as an autonomous decision-maker, and this makes explanation, not just prediction, essential [39,200,201,204,213].

Finally, implementation raises broader questions of fairness, governance, and responsibility. Training datasets may under-represent pediatric recipients, minority ethnic groups, and patients treated outside high-resource centers, creating the risk that AI tools will preferentially benefit populations that already receive better care. Reviews of AI-driven kidney allocation and broader transplantation AI repeatedly emphasize the need for transparency, fairness constraints, stakeholder oversight, and clear accountability frameworks. In parallel, recent discussion in healthcare AI has highlighted a practical tension that is especially relevant to transplantation: clinicians must not only know when to trust a model, but also when to override it [211,212,213,214].

Taken together, current AI applications in kidney allograft rejection are best viewed as enabling technologies rather than mature clinical solutions. Their most realistic near-term role is to support risk stratification, standardize parts of biopsy interpretation, and integrate multimodal data into more structured decision-support pathways. Their longer-term value will depend less on algorithmic novelty than on external validation, interpretability, workflow integration, and demonstration that they improve patient-centered outcomes rather than merely predictive metrics.

Table 9 summarizes the main current applications of AI and ML in kidney allograft rejection, highlighting their principal clinical uses, major limitations, and current level of readiness for practice.

## 8. Toward an Integrated, Rejection-Focused, Precision-Medicine Pathway

**Figure 1:**
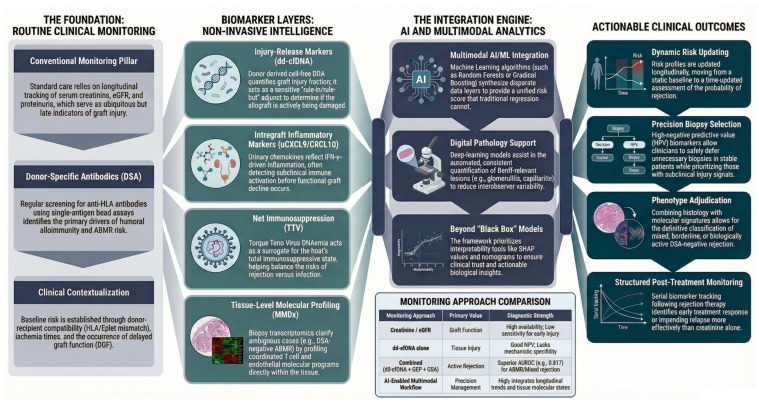
Proposed integrated workflow for rejection-focused precision medicine in kidney transplantation. Routine monitoring is complemented by biologically distinct biomarker layers, selective tissue adjudication, and multimodal integration, potentially supported by AI/ML. The aim is not biopsy avoidance in all cases, but earlier and more context-sensitive clinical decision-making.

The previous sections describe the building blocks; this section considers how they may be assembled into a coherent, rejection-focused precision-medicine pathway. The proposed workflow is summarized in Figure 1. The central idea is straightforward: move from a largely static approach based on eGFR, proteinuria, intermittent DSA testing, and occasional biopsy to a layered, dynamic system that updates an individual patient’s rejection risk over time using multiple orthogonal signals and links each risk state to a plausible clinical response [213].

Operationally, the proposed pathway can be understood as a four-step model rather than as a loose accumulation of tools. Step 1 is baseline biologic and clinical risk definition, integrating donor quality, HLA/eplet mismatch, sensitization history, delayed graft function, and drug-exposure context. Step 2 is longitudinal signal surveillance, in which serial biomarkers are interpreted according to their biological class: dd-cfDNA as an injury-release signal, urinary CXCL9/CXCL10 as inflammatory readouts, TTV as a marker of net immunosuppression, and blood GEP as a systemic immune-activation layer. Step 3 is tissue adjudication when circulating or urinary signals suggest that active injury or alloimmune activity may be present, particularly in patients with DSAs, rising biomarker trajectories, or unexplained graft dysfunction; in this step, histology and biopsy transcriptomics refine phenotype, distinguish mixed or DSA-negative cases, and reduce misclassification. Step 4 is post-diagnostic risk updating, in which biomarker trajectories, histology, molecular findings, and clinical response are reintegrated to determine whether the patient should return to routine surveillance, undergo intensified monitoring, or receive treatment adaptation. In this formulation, precision medicine is not a collection of parallel tests, but a working sequence in which each diagnostic layer answers a different biological question [24,150,215,216,217,218,219]. Framed this way, the pathway is intended to answer four sequential clinical questions: who starts in a high-risk biologic state; whether the evolving signal suggests injury, inflammation, or loss of immune control; when tissue adjudication is required to resolve phenotype; and whether subsequent trajectories support return to surveillance, continued close monitoring, or treatment adaptation. This is the practical distinction between a broad precision-medicine concept and a working rejection pathway.

A key conceptual shift is to define test relationships rather than accumulate uncoordinated tests. In other words, the value of precision medicine lies less in ordering more assays than in understanding what each assay contributes. dd-cfDNA is primarily an injury-release signal; urinary chemokines are closer to a readout of intragraft inflammation; TTV reflects net immunosuppression rather than rejection itself; tissue transcriptomics refines phenotype and molecular activity; and AI is most useful when integrating these heterogeneous layers rather than replacing any one of them. This distinction matters, because a precision pathway becomes biologically meaningful only when different signals are interpreted according to what they actually measure [150,173,220].

This also means that biomarker integration cannot be reduced to signal accumulation. A rising dd-cfDNA value, for example, should not be interpreted in the same way as an increase in urinary chemokines or a fall in TTV load, because these tests do not report the same biological event. The practical goal is not to force all signals into a single diagnostic label, but to use each of them according to its biological meaning and its main vulnerability to misclassification. In this framework, precision medicine becomes less a search for a perfect biomarker and more a structured method for reducing interpretative error.

At present, some elements of this pathway are already clinically plausible, whereas others remain investigational. Risk-adapted use of DSAs, dd-cfDNA, urinary chemokines, and selective biopsy is already feasible in advanced transplant programs. By contrast, routine use of multi-omic panels, AI-enabled longitudinal risk engines, and biomarker-driven immunosuppression adjustment remains more aspirational than established. Precision medicine in rejection will therefore need to evolve in stages, beginning with better integration of currently available tools before moving toward more complex, multi-layered systems [24,32,150,218].

Several barriers remain. Multi-omic integration still lacks robust harmonization pipelines and prospective evidence of incremental value over simpler biomarker combinations. Endpoint definition also remains a problem: broad outcomes such as “biopsy-proven rejection” or “doubling of creatinine” do not fully capture subclinical or chronic alloimmune injury, especially caABMR. As AI and ML become more deeply embedded in these pathways, governance, fairness, and continuous performance monitoring will become central rather than peripheral concerns [37,211,212,217,219].

A clinically plausible near-term scenario is therefore not biopsy avoidance in all cases, but better biopsy selection, earlier identification of rising alloimmune risk, and more structured post-treatment monitoring. For example, a recipient with stable eGFR but rising dd-cfDNA, increasing urinary chemokines, and a high-risk immunologic background might be triaged earlier to biopsy and tissue molecular profiling than would be the case under conventional surveillance alone. Conversely, a patient with stable biomarkers and low-risk longitudinal signals might safely avoid unnecessary tissue sampling. This is the level at which integrated precision medicine is most credible today: not replacing judgment but improving the timing and structure of it.

Finally, patient-centered outcomes must remain the anchor. Precision pathways are meaningful only if they reduce late graft loss, avoid unnecessary biopsies, and improve quality of life and equitable access to care. In that sense, rejection-focused precision medicine is less a single innovation than a gradual transition toward longitudinal, multimodal, and biologically informed decision-making [215].

## 9. Conclusions

Kidney allograft rejection remains a major barrier to durable transplant success despite excellent short-term outcomes. The field now has access to a growing set of tools, including non-invasive biomarkers, biopsy transcriptomics, and AI-supported analytical approaches, that can improve detection, phenotyping, and risk stratification [27,34,161,205]. The critical challenge is no longer simply to generate more signals, but to integrate them in ways that are biologically coherent, clinically interpretable, and actionable in routine care.

No single biomarker or model is sufficient on its own. dd-cfDNA reflects graft injury, urinary chemokines reflect inflammatory activity, DSAs define humoral risk, tissue transcriptomics refines phenotype, and AI can help combine these layers more effectively. The future of rejection-focused precision medicine therefore depends less on any single technology than on the intelligent coordination of complementary tools within risk-adapted care pathways.

In practical terms, the most credible near-term advance is a more selective and structured approach to surveillance, biopsy indication, and response monitoring, particularly in patients at higher risk of late alloimmune injury. Longer term, progress will depend on multicenter validation, better endpoint design, stronger implementation science, and careful attention to interpretability, equity, and patient-centered benefit. Precision medicine in kidney transplant rejection should therefore be judged not by how many tests it adds, but by whether it changes decisions early enough to alter the trajectory of chronic alloimmune injury and long-term graft survival.

## Figures and Tables

**Table 1:** Key distinguishing features of TCMR, ABMR, and mixed rejection.

Feature	TCMR	ABMR	Mixed Rejection
Dominant immune pathway	T-cell-mediated (direct, indirect, semidirect allorecognition)	Humoral alloimmunity driven by DSAs	Combination of cellular and humoral pathways
Histological hallmarks	Interstitial inflammation, tubulitis, ±intimal arteritis	Microvascular inflammation (g + ptc), C4d, transplant glomerulopathy	Coexistence of TCMR and ABMR lesions
DSA requirement	Not required	Usually present, but may be absent	Common, but not universal
Clinical behavior	May respond well to steroids (early episodes)	Often indolent, progressive	Worse prognosis than isolated TCMR or ABMR
Long-term impact	Risk of fibrosis and IF/TA if recurrent	Leading cause of death-censored graft loss	High risk of eGFR decline and transplant glomerulopathy

TCMR—T-cell-mediated rejection; ABMR—antibody-mediated rejection; DSAs—donor-specific antibodies; IF/TA—interstitial fibrosis and tubular atrophy; eGFR—estimated glomerular filtration rate.

**Table 2:** Epidemiology and clinical impact of rejection in contemporary cohorts.

Measure	Contemporary Estimate	Clinical Relevance
1-year biopsy-proven acute rejection	5–12%	Higher in sensitized and other high-risk recipients
Subclinical rejection	2.6–25%	Strongly dependent on surveillance biopsy protocols
ABMR cumulative incidence	1–21%	Increases overtime and contributes disproportionately to late graft injury
Predominant cause of death-censored graft loss	caABMR	Reflects the shift from early cellular rejection to chronic humoral injury
Rejection-attributed death-censored graft loss	47.5% overall; chronic ABMR 37.4%	Highlights the central contribution of late alloimmune injury
Predictors of dnDSAs/late alloimmune risk	DQ mismatch, tacrolimus intrapatient variability	Useful for risk-adapted surveillance

ABMR—antibody-mediated rejection; caABMR—chronic active antibody-mediated rejection; dnDSAs—de novo donor-specific antibodies.

**Table 3:** Strengths and limitations of conventional diagnostic tools.

Tool	Strengths	Limitations
Creatinine/eGFR	Widely available; longitudinal follow-up	Late marker; poor specificity; limited early detection
Proteinuria	Correlates with chronic injury	Nonspecific; affected by multiple conditions
DSAs (IgG SAB assays)	Predicts ABMR and graft loss	Prozone effect; interlaboratory variability; DSA-negative ABMR
Complement-binding assays	Adds risk stratification	Not diagnostic alone; assay variability
Biopsy	Gold standard; defines phenotype and Banff category	Sampling error; interobserver variability; invasive
C4d staining	Evidence of complement activation	May be negative in biologically active ABMR
Standard histology	Required for Banff classification	Subjective thresholds: patchy lesions, limited longitudinal integration

eGFR—estimated glomerular filtration rate; DSAs—donor-specific antibodies; SAB—single antigen beads, ABMR—antibody-mediated rejection.

**Table 4:** Overview of emerging non-invasive biomarkers for rejection.

Biomarker	Biofluid	What It Measures	Strengths	Limitations	Evidence Base
dd-cfDNA	Plasma	Donor-derived fraction reflecting graft injury	Good rule-in/rule-out performance; multicenter evidence	Affected by infection, biopsy, timing; platform variability	A
CXCL9/CXCL10	Urine	IFN-γ–inducible chemokines	High NPV; reflects intragraft inflammation	Influenced by UTI, BK virus, proteinuria	B
Gene-expression profiling	Blood	T-cell and IFN-γ–related signals	Complements dd-cfDNA; improves triage	Limited availability; cost	B
Proteomic panels	Serum/urine	Protein biomarkers	Mechanistic depth	Early phase; need multicenter validation	C
FTIR spectroscopy	Serum	Global biochemical fingerprint	High accuracy; low cost; rapid	Needs standardization and external validation	C

dd-cfDNA—donor-derived cell-free DNA; CXCL9—chemokine C-X-C motif ligand 9; CXCL10—C-X-C motif chemokine ligand 10; IFN-γ—interferon gamma; NPV—negative predictive value; UTI—urinary tract infections; FTIR—Fourier-transform infrared. Evidence base in this review: A, prospective multicenter validation and/or meta-analytic support; B, multicenter observational or embedded-validation evidence; C, single-center, early validation, or proof-of-concept evidence.

**Table 5:** dd-cfDNA application in rejection diagnosis.

Biofluid Type	Analyte/Assay (Technique)	Population & Dimensions	No. of Centers/ Studies	Prediction Models Features	References
Plasma	dd-cfDNA (% donor fraction; targeted NGS)	Observational, population-based; Europe + US; enrolled 2882, primary analysis n = 1134; external, transatlantic	14 centers	Threshold bands; incremental value over SOC (DSAs, labs) to detect active rejection	[32]
Whole blood + plasma	Blood GEP (qPCR panel) plus dd-cfDNA (Viracor-TRAC)	Prospective, “real-life” (for-cause + protocol biopsies); n = 230; Spain; blinded central lab; embedded validation	6 centers	Combined context-of-use: high TRAC + DSAs predict ABMR/mixed or MVI (AUROC 0.817); single tests underperform	[175]
Plasma	dd-cfDNA (NGS)	Pilot multicenter Japanese living-donor cohort; subclinical detection; feasibility	Multicenter pilot	Rule-in adjunct for surveillance; supports population-specific thresholds	[176]
Plasma	Total cfDNA, fractional dd-cfDNA, absolute dd-cfDNA (NGS/qPCR)	49 adult kidney transplant recipients (prognostic allograft dysfunction)	1 center	Models comparing total cfDNA vs. fractional/absolute dd-cfDNA to predict events	[177]
Meta-analysis	dd-cfDNA Several platforms	Systematic review post-2017 to 2023/24; n = 1248 patients	11 studies	Pooled accuracy for rejection (TCMR/ABMR); discusses cut-offs and heterogeneity	[31]

dd-cfDNA—donor-derived cell-free DNA; NGS—next-generation sequencing; SOC—standard of care; DSAs—donor specific antibodies; GEP—gene expression profiling; qPCR—quantitative polymerase chain reaction; ABMR—antibody-mediated rejection; MVI—microvascular inflammation; TCMR—t-cell-mediated rejection.

**Table 6:** Urinary chemokines (CXCL9/CXCL10), application in rejection diagnosis.

Biofluid Type	Analyte/Assay (Technique)	Population & Dimensions	No. of Centers/ Studies	Prediction Models Features	References
Urine	CXCL9, CXCL10 (automated immunoassays/ELISA; creatinine-indexed)	559 biopsy-paired urinary samples from 622 kidney transplants. Implementation paper defining context-of-use, pre-analytics, normalization; diagnostic accuracy for BPAR with clinical integration	Multi-lab, implementation	High NPV to rule out active rejection; emphasizes algorithmic use	[180]
Urine	CXCL9, CXCL10 (ELISA, with clinical covariates)	1082 samples collected at the time of a for-cause (71%) or a surveillance (29%) biopsy. Pragmatic model to guide biopsy decisions; includes validation and an open-access calculator	Multicenter cohort	Multivariable model (uCXCL9/10 + covariates) outperforms single thresholds	[26]
Urine	CXCL10 (ELISA; absolute vs. creatinine ratio)	Review paper; prognosis for dysfunction/rejection	Review paper	Absolute uCXCL10 is associated with subsequent injuries; supports serial trends	[29]
Urine	CXCL9/cre, CXCL10/cre (ELISA; linked to Banff scores)	117 urine samples. Cross-sectional + survival analysis; association with MVI, tubulitis, and graft survival	Single-center	Higher chemokines with higher i/ptc; elevated levels predict worse survival	[27]
Urine	CXCL9/CXCL10 (assorted platforms)	733 kidney transplants. Evaluation of incremental value beyond standard of care (labs + DSAs) for noninvasive detection	Prospective	Formal test of added value beyond SOC in real-world settings	[178]

CXCL9—chemokine C-X-C motif ligand 9; CXCL10—C-X-C motif chemokine ligand 10; BPAR—biopsy-proven acute rejection; NPV—negative predictive value; MVI—microvascular inflammation; SOC—standard of care.

**Table 7:** Other urine/blood biomarkers, application in rejection diagnosis.

Biofluid Type	Analyte/Assay (Technique)	Population and Dimension (Validation?)	Prediction Models (Features)	Evidence Base	Reference
Whole blood	GEP (qPCR panel), often paired with dd-cfDNA	“Real-life” mixed clinical + subclinical cohorts; multi-site; embedded validation	Combined GEP + dd-cfDNA improves noninvasive triage to biopsy vs. a single test	B	[181]
Plasma	TTV DNAemia (qPCR)	Systematic review/meta-analysis of transplant studies (kidney subset)	Risk-stratification for immunosuppression; modest AR discrimination; use trajectories	A	[182]
Plasma	TTV DNAemia (qPCR)	Contemporary transplant perspective; practice-oriented	Trajectory-based guidance for over/under-immunosuppression	C	[194]
Urine	Proteomics/peptidomics (micro-LC–TOF-MS)	4 European centers; n ≈ 311; discovery with confounder analysis	Multipeptide signatures for injury states; emphasis on standardization and external validation	B	[187]
Serum	Targeted proteomics panel (SAA1, AHSG, IGFBP2; DIA-MS → targeted)	KT recipients; discovery + internal validation	3-protein panel classifies acute rejection; requires multi-center external validation	C	[186]
Serum	FTIR spectral fingerprint (label-free)	n = 28 sera matched to biopsy; LOOCV; discovery	Naïve Bayes; AUC > 0.984 (cellular rejection vs. no rejection); key wavenumbers	C	[167]
Serum	FTIR + ML (label-free)	41 recipients/81 sera matched to biopsies; retrospective; expanded phenotyping	Naïve Bayes; AUC 0.945 (rej vs. no-rej) and 0.989 (TCMR vs. ABMR); feature-selected bands	C	[78]

GEP—gene expression profiling; qPCR—quantitative PCR; dd-cfDNA—donor-derived cell-free DNA; TTV—torque teno virus; AR—acute rejection; LC–TOF-MS—liquid chromatography–time-of-flight mass spectrometry; FTIR—Fourier-transform infrared; LOOCV—leave-one-out cross-validation; AUC—area under the curve; ML—machine learning. Evidence base in this review: A, prospective multicenter validation and/or meta-analytic support; B, multicenter observational or embedded-validation evidence; C, single-center, early validation, or proof-of-concept evidence.

**Table 8:** Biopsy transcriptomics/molecular microscope application in rejection diagnosis.

Biofluid Type	Analyte/Assay (Technique)	Population & Dimensions	No. of Centers/ Studies	Prediction Models Features	References
Biopsy tissue	Molecular microscope transcriptomics (microarray/RNA-seq classifiers)	Contemporary methods + evidence overview with kidney focus; summarizes classifier training/validation and clinical alignment with outcomes	Review (research-focused)	Clarifies how molecular archetypes map to rejection phenotypes and prognosis; use cases when histology is equivocal	[195]
Biopsy tissue	Molecular microscope longitudinal activity scores	Prospective/real-world evaluation with serial biopsies; activity tracking beyond morphology	Multi-site program	Continuous molecular scores reveal sub-threshold activity and track treatment response/relapse	[20]
Biopsy tissue (multi-center)	Molecular readouts alongside Banff histology (real-world implementation audit)	474 biopsies, August 2022–May 2024; implementation variability study	10 centers	Shows intercenter variability in indications, pipelines, and reconciliation of molecular vs. histology; calls for SOP harmonization	[165]
Biopsy tissue + plasma	Integrated tissue profiling linked to plasma dd-cfDNA	Cohort analysis mapping circulating injury signal to tissue molecular states	Cohort study	Elevated dd-cfDNA corresponds to three distinct molecular states (ABMR, recent parenchymal injury, TCMR); supports pairing blood and tissue signals	[197]
Biopsy tissue	Molecular microscope vs. histology in challenging patterns	326 consecutive biopsies; single-center study of discordance/limits	1 center	Highlights limits of molecular calls in isolated tubulitis/arteritis; reinforces clinicopathologic correlation	[169]
Biopsy tissue + blood	“Test relationships” framework (molecular, histology, DSAs, dd-cfDNA)	Contemporary integrated analysis	Multi-site (analysis)	Shows informative disagreements; dd-cfDNA/DSAs often align with molecular calls; proposes practical weighting rules	[196]
Biopsy tissue	Molecular endpoints within treated ABMR (trial-linked analysis)	Open-access biopsy transcriptomics captured during/after therapy	Multicenter trial context	Pathway-level profiles map response and relapse; supports molecular endpoints as complements to histology	[198]

dd-cfDNA—donor-derived cell-free DNA; ABMR—antibody-mediated rejection; TCMR—t-cell-mediated rejection; DSAs—donor-specific antibodies.

**Table 9:** Main current applications of artificial intelligence in kidney allograft rejection.

Application Domain	Main Input Data	Main Clinical Objective	Potential Strengths	Main Limitations	Current Readiness
Risk prediction for rejection and graft failure	Clinical, laboratory, and longitudinal follow-up data	Predict rejection, graft loss, and long-term outcomes	Handles complex interactions; supports individualized risk stratification	Mostly retrospective; limited external validation; center-specific bias	Early translational
Prediction of delayed graft function	Perioperative variables, donor/recipient data, biopsy features	Identify early post-transplant risk	Relevant early application; may support tailored monitoring	Not rejection-specific; validation remains limited	Early translational
Digital pathology and lesion quantification	Whole-slide images and annotated biopsy data	Support rejection detection and lesion scoring	May reduce interobserver variability; useful as second-reader support	Dependent on annotation quality; limited standardization; black-box concerns	Adjunctive only
Automated Banff-aligned lesion assessment	Whole-slide images with lesion-level annotations	Quantify Banff-relevant lesions more consistently	Closer to pathology workflow; potentially more interpretable	Requires high-quality labels; intercenter variability persists	Promising, not standardized
Multimodal biomarker integration	dd-cfDNA, urinary chemokines, GEP, DSAs, biopsy, clinical data	Improve triage and dynamic risk stratification	Combines orthogonal signals; aligns well with precision medicine	Data harmonization and validation remain limited	Promising, not routine
Allocation and donor–recipient matching support	Donor, recipient, immunologic, and outcome datasets	Improve matching and allocation efficiency	Integrates many variables simultaneously	Fairness, transparency, and governance concerns	Experimental
Clinical decision support in follow-up	EHR, drug exposure, biomarkers, biopsy history	Guide monitoring and biopsy decisions	May improve workflow and structured follow-up	Requires interoperability, explainability, and clinician trust	Immature

## Data Availability

Not applicable.

## Abbreviations

The following abbreviations are used in this manuscript:

caABMR	Chronic active antibody-mediated rejection
DSAs	Donor-specific antibodies
HLA	Human leucocyte antigens
ABMR	Antibody-mediated rejection
KT	Kidney transplantation
TCMR	T-cell–mediated rejection
eGFR	Estimated glomerular filtration rate
dd-cfDNA	Donor-derived cell-free DNA
AI	Artificial intelligence
ML	Machine learning
MVI	Microvascular inflammation
Ptc	Peritubular capillaritis
BPAR	Biopsy-proven acute rejection
dnDSAs	de novo DSAs
EV	Extracellular vesicles
DGF	Delayed graft function
KDPI	Kidney Donor Profile Index
KDRI	Kidney Donor Risk Index
IRI	Ischemia–reperfusion injury
FTIR	Fourier-transform infrared spectroscopy
SAB	Single-antigen bead
SOC	Standard of care
GEP	Gene expression profiling
TTV	Torque teno virus
